# Incorporation of Fully Bio-Based Butylene Succinate Oligomers into Poly(butylene succinate) by Melt Mixing

**DOI:** 10.3390/polym18101190

**Published:** 2026-05-13

**Authors:** Carmen Olivas Alonso, Amparo Chiralt, Sergio Torres-Giner

**Affiliations:** University Institute of Food Engineering—FoodUPV, Universitat Politècnica de València (UPV), Camino de Vera s/n, 46022 Valencia, Spain; carolal1@etsii.upv.es (C.O.A.); dchiralt@tal.upv.es (A.C.)

**Keywords:** oligomer, poly(butylene succinate), biopolymer formulations, food packaging

## Abstract

In this study, fully bio-based oligomers of butylene succinate (OBS) with different molecular weights (low: L-OBS, medium: M-OBS and high: H-OBS) were incorporated into poly(butylene succinate) (PBS) by melt mixing at varying loadings of 5–15 wt%. Then, PBS/OBS films were obtained by thermo-compression and characterized to assess their suitability for sustainable food packaging. Thus, OBS were homogeneously incorporated into PBS matrix and modulated the thermal, mechanical, and barrier properties of the PBS. L-OBS (Mn = 1150 g·mol^−1^) plasticized the amorphous PBS, depending on its concentration, more effectively than M-OBS (Mn: 8700 g·mol^−1^) and H-OBS (Mn: 18,650 g·mol^−1^), as deduced from the thermo-mechanical analysis. In every case, OBS enhanced crystallinity, mainly L-OBS, which reduced the film strength and increased water vapor permeability, depending on its concentration. In contrast, H-OBS improved mechanical strength, stiffness, and barrier performance. In all cases, X-ray diffraction confirmed the preservation of the PBS’s monoclinic crystalline structure but slightly shifted the diffraction angle depending on the ratio of the end-chain groups in the blend, thus reflecting the contribution of OBS in the crystalline lattice. Finally, oligomer incorporation resulted in an overall migration increase in different food simulants, impairing their application in packaging.

## 1. Introduction

The accumulation of plastic packaging waste in both marine and terrestrial environments has become a critical global concern, prompting the search for sustainable materials that mitigate reliance on non-renewable resources and reduce environmental impact. Biodegradable plastics derived from renewable biomass have thus acquired substantial interest as viable alternatives to conventional packaging polymers. These materials align with worldwide efforts to enable a low-carbon economy by decreasing dependence on fossil fuels and diminishing the environmental impact of plastic accumulation [1].

In particular, biopolyesters synthesized from renewable succinic acid—including poly(butylene succinate) (PBS) and its copolymers—are among the most promising candidates for replacing traditional polyolefins due to their processability and physical properties [2]. PBS is a semicrystalline aliphatic polyester produced via esterification and polycondensation reactions between succinic acid (SA) and 1,4-butanediol (BDO), which can be both synthesized from fully renewable sources [3]. PBS is biodegradable and it demonstrates good thermoplastic processability and well-balanced mechanical properties. Additionally, PBS is authorized by the U.S. Food and Drug Administration (FDA) for certain food-contact applications under FCN No. 1818 [4]. However, certain improvements, such as low-viscosity processing, higher mechanical strength and flexibility, and barrier performance, must still be addressed to enable broader adoption in food packaging [5].

Polymer performance depends not only on intrinsic properties, but also on the processing methods—including extrusion, injection molding, foaming, and thermoforming—as well as on modifications, such as copolymerization, blending, surface treatment, and the addition of fillers or additives [6,7,8]. Among these, the incorporation of bio-based additives is particularly relevant in the development of biodegradable packaging with tailor-made properties, for instance, processing aids and enhancers in the mechanical and barrier performance or modifiers of aesthetic characteristics, among others [9,10,11,12,13]. In relation to PBS and its copolymers, some earlier studies have investigated a broad range of additives, such as unmodified and organo-functionalized clays [14] or inorganic compounds (e.g., zeolite) [15]. In a recent study, Chiu et al. [16] investigated the incorporation of halloysite nanotubes (HNTs) and organo-modified montmorillonite (OMMT) into poly(butylene succinate-*co*-adipate) (PBSA) to fabricate bio-based nanocomposites with enhanced mechanical performance for potential use in rigid packaging. The inclusion of 5 wt% OMMT or HNTs with good dispersion of both nanofillers led to a marked increase in the storage modulus of up to 117%. In another study, blends of PBS with polylactide (PLA) were reinforced with iron-functionalized zeolite to enhance barrier properties (36%) for fruit packaging applications [15]. In another study, Tachibana et al. [17] investigated the addition of cellulose acetate butyrate (CAB) as a multifunctional additive to PBS via melt blending. The incorporation of 10 wt% CAB significantly increased PBS flexibility (strain at break up to 547%) without reducing tensile strength (36 MPa). However, the addition of CAB drastically reduced the biodegradability of PBS under composting conditions, suggesting CAB can also act as a biodegradation inhibitor, influencing the crystalline structure of PBS. Furthermore, the additive manufacturing of biodegradable hemp-reinforced PBS filaments was explored using fused filament fabrication (FFF) [18]. Two types of hemp fibers (short ≤ 2 mm and long ≤ 10 mm) were incorporated at 5 wt%, improving the PBS stiffness by up to 63%.

As polymer additives, oligomers have demonstrated considerable potential by improving processability [19] and/or conferring tailored physical properties [20]. Following this strategy, different oligomers have been incorporated into poly(ε-caprolactone) (PCL) such as low-molecular-weight PCL [19] or oligoesters of poly(glycerol-succinate) [20]. In the first study, blending low- and high-molecular-weight PCL accelerated degradation, improved processability, and reduced polymer crystallinity of electrospun fibers [19]. In the second one, bio-sourced oligoesters based on poly(glycerol-succinate) were successfully well dispersed in the PCL matrix, modifying its crystallization and accelerating degradation [20]. Similar effects have been reported in other polyester systems. Lim et al. [21] investigated the effect of PCL molecular weight (Mw: 530–80,000 Da) in PET/PCL blends (35 wt% PCL). The incorporation of low-molecular-weight PCL led to a decrease in the crystallization temperature (Tc) of PET, indicating increased chain mobility and disruption of the polymer matrix. Furthermore, low-Mw PCL (Mw: 1250 and 530 Da) exhibited a higher reactivity towards PET chains, attributed to their greater mobility and higher concentration of hydroxyl end groups.

In parallel, extensive research has been conducted on lactic acid oligomers (OLAs). Burgos et al. [22] incorporated 15% OLAs with different Mw (860–1350 Da) into PLA and observed a decrease in its glass transition temperature (T_g_) and an increase in the film extensibility. Ambrosio-Martín et al. [23] observed similar effects for OLAs on the thermal behavior of PLA, with an improvement of oxygen barrier capacity but no remarkable increase in the film extensibility. Yang et al. [24] further investigated blends of high- and low-molecular-weight PLA (HPLA and LPLA, Mw ≈ 2100 and 90 Da, respectively). The addition of LPLA led to a slight decrease in T_g_ and crystallinity, indicating a dilution effect of the low-MW fraction. Additionally, the reduction in tan δ suggested increased chain mobility and good miscibility, while tensile strength decreased and toughness was moderately enhanced. Schliecker et al. [25] examined the effect of OLAs with different molecular weights (Mw ≈ 1700 and 3200 Da) in polylactide-*co*-glycolide (PLGA) films at concentrations of 0–30 wt%. Oligomer incorporation reduced T_g_ of the polymer while promoting earlier crystallization and increasing crystallinity, particularly at higher contents and lower molecular weight oligomers. Avolio et al. [26] also analyzed the effect of two oligomers of types of OLAs, namely carboxyl (OLA-COOH) or hydroxyl (OLA-OH) end-capped (Mn: 1050–1180 Da), on the physical and mechanical properties of PLA at concentrations of up to 25 wt%. Both the T_g_ and onset thermodegradation rate decreased as the OLA concentration rose, while film extensibility increased only from concentration levels of 20 wt% for both types of OLAs. Migration tests also revealed that the overall migration values remained below the legal limit only under conditions where the PLA amorphous phase remained glassy. In this regard, one should consider that oligomers can more easily release into packaged products, raising food safety concerns, depending on their molecular weight, concentration, and toxicity [27].

In our recent study, fully bio-based oligomers of butylene succinate (OBS) with weight-average molecular weight (M_w_) values ranging from 2050 g·mol^−1^ to 33,147 g·mol^−1^ were successfully synthetized and characterized [28]. These oligomers were obtained by controlling the conditions and extension of a previously developed multi-step polycondensation process, using renewable SA and BDO [3]. To the best of our knowledge, no previous studies have specifically reported the OBS incorporation into PBS. Therefore, significant knowledge gaps remain in the effect of OBS on the processability and properties of PBS. In the present study, fully bio-based OBS samples with different molecular weights were incorporated at different ratios into PBS by melt mixing, and the resultant materials were thermo-compressed into films to evaluate their microstructure, thermal stability and phase transitions, crystallinity, mechanical yield and oxygen and water vapor barrier properties. Finally, the global migration of the PBS/OBS films was analyzed in different food simulants to ascertain their safety in food packaging applications.

## 2. Materials and Methods

### 2.1. Materials

PBS was supplied, in the form of pellets, by PTT MCC Biochem Company Limited (Mitsubishi Chemical Corporation, Tokyo, Japan) as BioPBS FZ91PM. This polyester grade is partially bio-based from renewable succinic acid. It has a density of 1.26 g·cm^−3^ and it is designed to be intended to come in contact with food, since it complies with EU No. 10/2011 and is also biodegradable under industrial composting conditions with the “OK Compost” certification (EN 13432).

Acetic acid was supplied by Labkem (Labbox Labware S.L., Barcelona, Spain), while magnesium nitrate (Mg(NO_3_)_2_), di-phosphorus pentoxide (P_2_O_5_), ethanol and isooctane were supplied by Panreac Química S.L.U. (ITW Reagents Panreac, Castellar del Vallès, Barcelona, Spain).

### 2.2. Oligomer Synthesis

OBS was synthesized by polycondensation reaction of renewable SA with BDO in two steps, namely esterification and transesterification, following the conditions reported in our previous study [28]. The reaction was conducted in a 100-mL glass-jacketed reactor connected to a Huber Pilot Pilot ONE CC-304B immersion bath (Peter Huber Kältemaschinenbau AG, Offenburg, Germany) for precise temperature control and equipped with a mechanical stirrer (Heidolph RZR-2021 rotor, Heidolph Instruments GmbH & Co. KG, Schwabach, Germany) and a Vigreux condenser column with a glass distillation adapter. During the esterification step, short-chain oligomers were formed by the reaction of SA with excess BDO, whereas in the catalytic transesterification, the molecular weight of the oligomers was increased by the addition of the catalyst titanium (IV) isopropoxide (TTiP) followed by the application of heat (up to 230 °C) and high vacuum (<1 mbar). Further details of this oligomerization/polymerization unit can be found in the literature [22]. Three different types of oligomers were produced: OBS-L with a low molecular weight that was obtained at the end of the esterification, OBS-M with medium molecular weight, obtained after 1 h of transesterification, and OBS-H with high molecular weight, obtained after 2 h of transesterification. Table 1 gathers the values of number- and weight-average molecular weight (M_n_, M_w_), and dispersity (Ð) of the commercial PBS and synthesized oligomers, all determined by gel permeation chromatography (GPC) using HR5E and HR2 Waters linear Styragel columns (7.8 × 300 mm, pore 10^3^–10^4^ Å and refractive index (RI) and ultraviolet (UV) detectors (Waters Cromatografía, S.A, Cerdañola del Vallés, Spain).

### 2.3. Film Production

The PBS pellets were first dried at 70 °C for 2 h in a vacuum oven (Model 207, J.P. SELECTA, S.A., Barcelona, Spain). The OBS samples were cryogenically ground in a milling device, Thermomix TM6 (Vorwerk, Wuppertal, Germany), under liquid nitrogen and then vacuum-dried at 50 °C for 2 h. All samples were thereafter transferred to a desiccator containing P_2_O_5_ (0% relative humidity, RH) and stored for 24 h to remove residual moisture, minimizing biopolyester hydrolysis during thermo-mechanical processing.

Melt mixing was conducted in an internal mixer equipped with a dual-rotor system HAAKE PolyLab QC (Thermo Scientific, Waltham, Massachusetts, USA) at 150 °C and 50 rpm for 5 min. Each oligomer (OBS-H, OBS-M, and OBS-L) was blended with PBS at 5, 10, and 15 wt%. The same procedure was applied to neat PBS to prepare a control sample with an identical thermo-mechanical history. Table 2 gathers the different PBS/OBS formulations prepared, which were designated as Hx, Mx, or Lx, where H, M, and L correspond to the use of OBS-H, OBS-M, and OBS-L, respectively, and x indicates the oligomer content. The resultant doughs were cryogenically milled in the Thermomix TM6 device and dried in a desiccator with P_2_O_5_ (0% RH) for 48 h prior to film processing.

Finally, the dried powders were thermo-compressed in a hydraulic press (Model LP20, Labtech Engineering Co., Ltd., Samutprakarn, Thailand). The process consisted of a preheating at 150 °C for 5 min, followed by compression at 150 °C and 100 bar for 4 min. The resulting control PBS and PBS/OBS films were approximately 200-μm thick (see Table 2). Prior to analysis, the films were conditioned at either 0% or 53% RH, depending on the test requirements.

### 2.4. Characterization

#### 2.4.1. Melt Flow Rate

Melt flow rate (MFR) was determined according to ISO 1133 using a PCE-MFI 400 plastometer (PCE Instruments, Meschede, Germany) under a standard load of 2.16 kg. The material was introduced into the preheated barrel and allowed to equilibrate prior to measurement. Neat PBS was initially tested at 190 °C, following standard conditions and the manufacturer’s recommendations. However, due to the high fluidity of PBS/OBS formulations—particularly those containing OBS-L—reliable measurements at this temperature were not feasible. Therefore, MFR measurements were performed at 150 °C for all samples to ensure consistent and comparable results. All measurements were carried out in duplicate, and the results are reported as mean values with standard deviation.

#### 2.4.2. Microstructural Analysis

The internal structure of the PBS/OBS film cross-sections was examined by FESEM. To this end, the samples were cryogenically fractured by immersing them in slush nitrogen and then coated with platinum using an EM MED020 sputter coater (Leica BioSystems, Barcelona, Spain). Subsequently, images were captured in a FESEM equipped with a focused ion gun (Auriga Compact, Carl Zeiss SMT, Oberkochen, Germany) at 2.0 kV and a magnification of 14,000×.

#### 2.4.3. Thermogravimetric Analysis

The thermal stability of the PBS/OBS films was determined by thermogravimetric analysis (TGA) using a thermogravimetric analyzer (Mettler-Toledo Inc., TGA 1 Stare System analyzer, Schwerzenbach, Switzerland) in the thermal range of 25 °C to 700 °C at a heating rate of 10 °C·min^−1^ under nitrogen flow (10 mL/min). The TGA and first derivative (DTGA) curves were attained using a STARe Evaluation Software (Mettler-Toledo Inc.).

#### 2.4.4. Differential Scanning Calorimetry

The phase transitions of the PBS/OBS films were analyzed by differential scanning calorimetry (DSC) in a DSC823^℮^ Star^℮^ (Mettler-Toledo Inc.), operating under a nitrogen flow (10 mL·min^−1^). Samples (5–6 mg) were placed into aluminum pans and tightly sealed. Then, these were placed in the calorimeter, and the following three-step program was applied: a first heating from −40 to 150 °C to erase the thermal history, followed by cooling to −40 °C, and then a second heating to 150 °C. As a reference sample, an empty aluminum pan was used. Each sample was analyzed in duplicate, using heating and cooling rates of 10 °C·min^−1^.

#### 2.4.5. X-Ray Diffraction Analysis

Crystallinity of the PBS/OBS films was also studied with an X-ray diffractometer (AXS/D8 Advance, Bruker, Karlsruhe, Germany). Wide-angle X-ray diffraction (WAXD) diffractograms were obtained at 40 kV and 40 mA, with a step size of 0.04°·min^−1^ and 2θ scanning angle between 5° and 50°. The film samples, previously conditioned at 0% RH, were mounted tightly and flush with the surface of the XRD sample holder to ensure proper alignment and reduce background noise. Data were obtained using a 1D LynxEye detector with XRD Commander software (Bruker AXS GmbH, Karlsruhe, Germany) and processed with Match! software (version 2.0, Crystal Impact, Bonn, Germany). The crystallinity values (*χ*_c_) were calculated using Equation (1) by deconvolution of the amorphous halo, using the OriginPro 2026 (OriginLab Corporation, Northampton, MA, USA) [29], where *Ac* and *A_a_* represent the crystalline and amorphous areas, respectively.

(1) $$\chi_{c}=\left(\frac{A_{c}}{A_{c}+A_{a}}\right)\cdot 100$$

The lattice parameters (a,b,and c) and the monoclinic angle (β) were calculated considering that the materials exhibit the diffraction peaks corresponding to the monoclinic crystal lattice of PBS at α-form [30]. Bragg’s Law (Equation (2)) was applied to obtain the values of interplanar spacing (*d_hkl_*), where n=1 is the order of diffraction, λ is the wavelength of the incident radiation (1.542 Å for CuK_α_ radiation), and *θ* is the angle between the incident X-ray beam and each respective crystal plane:

(2) $$n\cdot \lambda =2\;{\cdot d}_{hkl}\cdot sin(\theta )$$

Thereafter, Equation (3) was used as the general equation in a monoclinic crystal system to determine the lattice parameters and angle for each interplanar distance [31], where h,k, and l are the Miller indices for each specific diffraction peak. Thus, a system of nonlinear equations was constructed from the *d_hkl_* values of the first four characteristic diffraction peaks. A Python code (Python version 3.11.10) was developed using the NumPy v2.0.1 library to solve the system of equations based on the input of the four interplanar distances associated with the four main crystalline peaks in the XRD analysis.

(3) $$\frac{1}{{d^{2}}_{hkl}}=\frac{h^{2}}{a^{2}\cdot {sin}^{2}\beta }+\frac{k^{2}}{b^{2}}+\frac{l^{2}}{c^{2}\cdot {sin}^{2}\beta }-\frac{2\cdot h\cdot l\cdot cos\beta }{a\cdot c\cdot {sin}^{2}\beta }$$

#### 2.4.6. Thermo-Mechanical Analysis

Dynamic mechanical thermal analysis (DMTA) was conducted on the PBS/OBS films using a Discovery DHR-1 oscillatory rheometer (TA Instruments, New Castle, DE, USA), equipped with a torsion clamp system for solid samples. Samples, sizing 5 mm × 15 mm, were heated from −50 to 150 °C at a rate of 2 °C·min^−1^, with a maximum deformation (γ) of 0.1%, maintaining a constant frequency of 1 Hz.

#### 2.4.7. Mechanical Test

Tensile tests were carried out at room temperature on the PBS/OBS films in accordance with the ASTM D638 standard, using a universal testing machine, equipped with a 500-N load cell (Shimadzu AGS-X, Kyoto, Japan), with a cross-head speed of 10 mm·min^−1^. Samples were previously die-cut into type IV specimens (width 6 mm, length 55 mm). At least seven samples per formulation were examined.

#### 2.4.8. Permeability Tests

Oxygen permeability (OP) was determined by following the ASTM standard method D3985 05 [32]. For this, films of 50 cm^2^ of each formulation were placed in an oxygen permeation analyzer (Model 8101e, Systech Illinois, IL, USA) at 25 °C and 53% RH. OP was calculated by dividing the oxygen transmission rate (OTR) by the difference in oxygen partial pressure between the two sides of the film and multiplying by the film thickness. The water vapor permeability (WVP) of the films was determined gravimetrically in accordance with ASTM E96/E96M (ASTM, 2005) [33] and the experimental procedure described by Hernández-García et al. [34]. The samples were cut and placed in circular Payne cups (Ø = 3.5 cm) containing 5 mL of distilled water (100% RH). Then, the cups were put into desiccators at 25 °C and 53% RH (with Mg(NO_3_)_2_ over-saturated solution). The cups were weighed every 2 h for 24 h, and the water vapor transmission rate (WVTR) was determined from the slope of the weight loss versus time curve and corrected for permeant partial pressure to yield permeance. WVP was finally determined by taking the film thickness into account. The measurements were taken in triplicate for each film.

### 2.5. Global Migration Test

Global Migration tests were conducted on the PBS/OBS film samples using five different simulants: distilled water (simulant A), 3% (wt/vol) acetic acid in water (simulant B), 10% (vol/vol) ethanol in water (simulant C), 95% (vol/vol) ethanol (simulant D), and isooctane (simulant E). Simulants A, B, and C were used to ascertain the film migration in aqueous food, while simulants D and E were used as substitutes for fatty foods. The tests were performed in accordance with the UNE-EN 1186-1:2002 standard. As specified in the standard, the migration test maintained the equivalence of 100 cm^2^ of film per 100 mL of food simulant in which the film is immersed. The selected conditions were as follows: 10 days at 40 °C in an oven (Model 207) for simulants A, B, C, and D, and 2 days at 20 °C in a climatic chamber (Hotcold UC, J.P. Selecta, S.A.) for simulant E. These conditions correspond to the standardized test conditions OM2, which cover the contact of the material with food during prolonged storage periods at ambient or lower temperatures. After the exposure time, the films were removed, and the simulants were evaporated to quantify the mass of solid residue corresponding to the overall migration (OM) of each film. All migration measurements were performed in duplicate.

### 2.6. Statistical Analysis

Results were submitted to analysis of variance (ANOVA) using Statgraphics Centurion XVII-64 software (Manugistics Corp., Rockville, MD, USA). Fisher’s Least Significant Difference at a 95% confidence level was used (*p* < 0.05).

## 3. Results and Discussion

### 3.1. Melt Mixing of PBS/OBS

**Figure 1:**
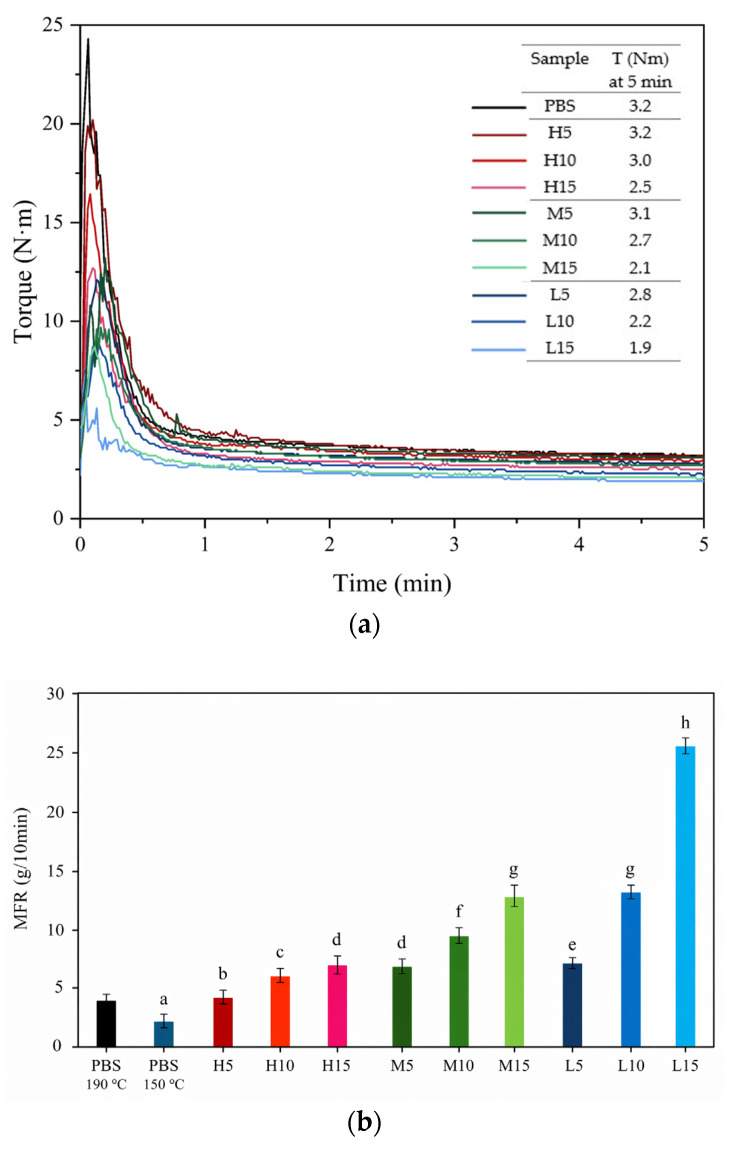
(**a**) Development of torque as a function of mixing time during the melt-mixing process of poly(butylene succinate) (PBS) with the different oligomers of butylene succinate (H, M, L series) at 5, 10, and 15 wt%. (**b**) Melt flow rate (MFR) values of the PBS and PBS/OBS films. Different letters indicate statistically significant differences (*p* < 0.05).

The processability of the PBS/OBS formulations was analyzed in Figure 1. Thus, Figure 1a shows the torque-vs-time curves obtained during the melt-mixing process for the neat PBS and the PBS/OBS compositions obtained with the oligomers of the three different molecular weights (H, M, L) at the three selected oligomer contents (5, 10, and 15 wt%). This was carried out, in all cases, at 150 °C and 50 rpm for 5 min. This residence time was selected to process all formulations in order to achieve sufficient mixing in the blends but also to avoid thermomechanical degradation [35].

In all formulations, a high increase in torque was observed at the onset of mixing, which reflects the energy required to plastically deform the PBS pellets and their transition from a solid-like material to a liquid in the molten state. Thereafter, as the biopolymer melted and became more fluid, the torque rapidly decreased, reaching a relatively stable plateau in the 3–4 N·m range that highlights the quasi-steady-state melt viscosity, which reflects an equilibrium among biopolymer chain relaxation and shear-induced orientation. Moreover, the relatively high stability of the torque in the plateau region suggests that no significant cross-linking or degradation reactions took place under applied processing conditions. The inset table shows the torque values reached after 5 min of melt mixing to reflect the plasticizing effect of the oligomers on PBS. For the neat PBS sample (see black curve), the higher torque profile curve indicates its relatively higher melt viscosity and lower melt fluidity compared to the OBS-containing samples. In contrast, the samples containing the oligomers consistently displayed lower torque values in their final mixing stages. These values decreased as the OBS content rose or its molecular weight decreased, reflecting the effect of the oligomer type and content on the melt viscosity. This effect can be regarded as a global reduction in the mean molecular weight of the melt, thereby enhancing chain mobility and lowering the resistance to flow [36]. Similar results were obtained by Yang et al. [24] during the processing of low-/high-molecular-weight PLA blends. Therefore, these torque profiles confirm that the incorporation of these bio-based OBS can effectively reduce the melt-flow characteristics of PBS, acting as processing aids since these can potentially offer easier processability and lower energy consumption during melt manufacturing.

Plasticization was further analyzed by the measurement of MFR values of the different OBS/PBS formulations. The results gathered in Figure 1b were coherent with the torque profile curves shown above, revealing a significant increase (*p* < 0.05) in the MFR values with both increasing the oligomer content and decreasing their molecular weight. Thus, neat PBS exhibited the lowest MFR values, that is, 3.7 and 1.8 g/10 min at 190 and 150 °C, respectively, whereas all PBS/OBS formulations showed significantly higher values (*p* < 0.05), consistent with the good miscibility of OBS in the matrix and the consequent reduction in the apparent molecular weight of the biopolymer formulation. Therefore, the incorporation of OBS reduced molecular interactions, leading to increased chain mobility and a consequent decrease in PBS melt viscosity. The effect was particularly pronounced for the L-series, which exhibited the highest MFR values at 15 wt% (25.7 g/10 min at 150 °C), indicating a strong reduction in melt flow resistance. Therefore, this confirms that OBS can act as effective flow promoters, enhancing the processability of PBS.

### 3.2. Microstructure of PBS/OBS Films

**Figure 2:**
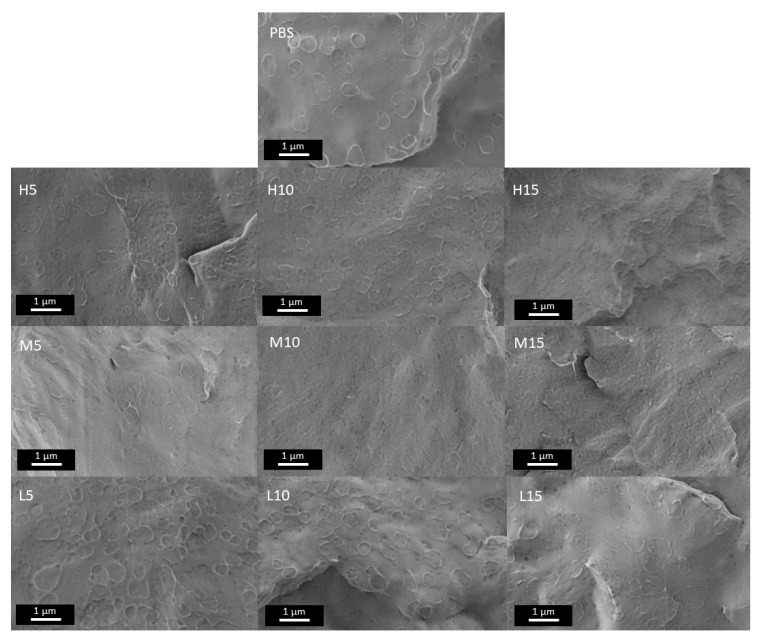
Cross-sectional fractures attained in cryogenic conditions of the films of poly(butylene succinate) (PBS) with the different oligomers of butylene succinate (H, M, L series) at 5, 10, and 15 wt%. Images taken at 14,000× magnification with a scale of 1 µm.

The cross-section surfaces of the neat PBS film and OBS-containing PBS films in all their contents and compositions are gathered in Figure 2. In all cases, the films exhibited the typical brittle fracture observed for PBS [37]. Incorporation of OBS at different ratios did not induce appreciable changes in the film microstructure and no separated phases were observed. This supports the good miscibility between PBS and OBS, which was also evidenced by the flow properties of the melt blends. Several irregularly sized rough edges were observed, likely corresponding to the PBS crystalline domains [37,38].

### 3.3. Thermal and Crystallization Behavior of PBS/OBS Films

TGA was performed on the PBS/OBS samples to analyze the effect of the oligomers on PBS. Table 3 includes the parameters determined for each thermal degradation event obtained from the TGA curves (Figure S1a) and their corresponding DTGA curves (Figure S1b). No significant (*p* > 0.05) weight loss occurred between 30 °C and 140 °C, confirming the lack of bound water and suggesting that no low-molecular-weight chains or oligomers volatilized. Significantly lower values (*p* < 0.05) were observed for the onset temperature associated with the 5% weight loss (T_5%_), used to determine the effect of the oligomers on the thermal resistance of the PBS films. Similar results were reported by Burgos et al. [22] for PLA films melt mixed with OLAs due to the lower thermal stability of low molecular weight compounds. Thus, neat PBS exhibited the highest values of T_5%_ (345 °C), according to the superior thermal stability of its high molecular weight structure. Coherently, among the PBS/OBS films, the samples containing H oligomers (H5, H10, and H15) demonstrated better thermal resistance than the samples produced with M and L oligomers, with T_5%_ values ranging between 266 and 280 °C. These values are consistent with the previously reported thermal resistance of OBS [28]. Interestingly, the PBS films prepared with the L-series oligomers showed higher T_onset_ values (262–268 °C) than the same films prepared with OBS-M. In this regard, the presence of the remaining amounts of the metallic catalyst added during the transesterification step, used for the synthesis of the H and M oligomers, may contribute to the observed decrease in thermal stability, as previously reported in the literature [39,40].

The values of the temperature of maximum degradation rate (T_peak_ in DTGA curves) were similar in all cases. Main thermal degradation took place in the 300–450 °C range, with a maximum degradation rate at T_peak_ of nearly 400 °C, which is consistent with the findings reported previously [41]. The second and low-intensity mass loss step, seen at the thermal range of 450–550 °C, can be attributed to the thermal decomposition of the organic compounds produced during the previous steps [41,42], being similar for all the film samples. Therefore, the thermal stability of the PBS films decreased due to the presence of the oligomers due to their low-molecular-weight chains; however, thermal degradation of PBS was seen to remain nearly unaltered.

**Figure 3:**
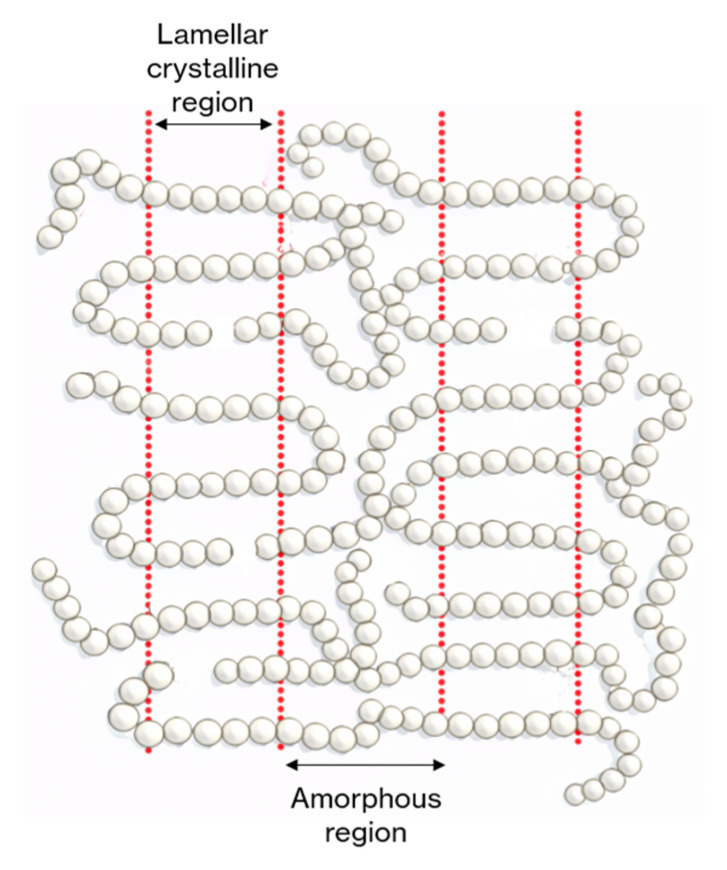
Schematic representation of the way end-chain groups of oligomers can interfere with the lamellar crystalline structure of the biopolymer matrix.

Table 4 shows the first-order thermal phase transitions, that is, crystallization temperature (T_c_) and melting temperature (T_m_), and their corresponding melting enthalpy ΔH_m_) values of the PBS/OBS films obtained from the DSC curves during first heating (Figure S2a), cooling (Figure S2b), and second heating (Figure S2c). The slight differences in the thermal parameters obtained in the first and second heating steps must be attributed to the thermal history of the samples. Data obtained in the second heating are more reliable due to the controlled cooling rate of the molten samples. During the first heating step, all samples exhibited a non-reversible (it did not appear in the second heating), low-intensity second-order transition, which has been previously ascribed to the transition of the rigid amorphous fraction (RAF) from solid-like RAF into liquid-like amorphous fraction, in the context of the multiple melting behavior of PBS [30]. All the samples exhibited a broad endothermic peak centered in the 112–115 °C range, corresponding to the T_m_ value of PBS. Nevertheless, biopolymer’s cold crystallization was also observed in the range of 100–105 °C, similar to that previously reported [30]. This behavior indicates the presence of a wide range of crystal sizes and/or multiple phenomena of melting/recrystallization phenomena in the PBS samples [30,43]. The incorporation of the oligomers did not alter this thermal behavior, but the values of T_m_ were slightly reduced in the samples with the low-molecular-weight oligomer (L series), whereas this trend was more difficult to observe for those containing the M- or H-type oligomers. This suggests the formation of less perfect lamellar crystals when short-chain oligomer molecules are present due to the higher ratio of end-chain groups. As schematically illustrated in Figure 3, these end-chain groups may interfere with the lamellar crystals, inducing more defects.

The cooling thermograms revealed the presence of a single exothermic peak with a T_c_ value of 85.2 °C for the neat PBS sample, which is similar to that previously reported [44]. The incorporation of the oligomers slightly reduced the T_c_ values from the melt, which was more evident in the case of the films with low-molecular-weight oligomers (L series) due to the supercooling increase with their content. Therefore, the presence of the oligomers with lower molecular weight delayed the formation of the PBS crystals, suggesting that these oligomers interfered with the biopolymer crystallization due to the afore-mentioned higher ratio of the end-chain groups in the macromolecule. A comparable behavior was reported by Lim et al. [21], who found that T_c_ decreased in PET melt mixed with PCL oligomers.

Values of ΔH_m_ were expressed per g of film, considering that oligomers can participate in the crystalline regions given the similar molecular structure. The ΔH_m_ values, and hence the crystallinity degree, increased in the OBS-containing films. This result indicates that, even though oligomers induced less ordered/stable crystalline forms, these promoted crystallization to a slightly different extent, depending on their molecular weight and ratio. This promotion was highest in the OBS-L, where ΔH_m_ values also increased with the oligomer content. This crystallization enhancement is consistent with the plasticizing effect and subsequent molecular mobility promotion of the PBS by OBS. In this regard, the previous study of Schliecker et al. [25] showed that OLAs promoted the crystallization of PLGA, while the degree of crystallinity was affected by both the average molecular weight and content of oligomer.

### 3.4. Crystallinity Analysis of PBS/OBS Films

**Figure 4:**
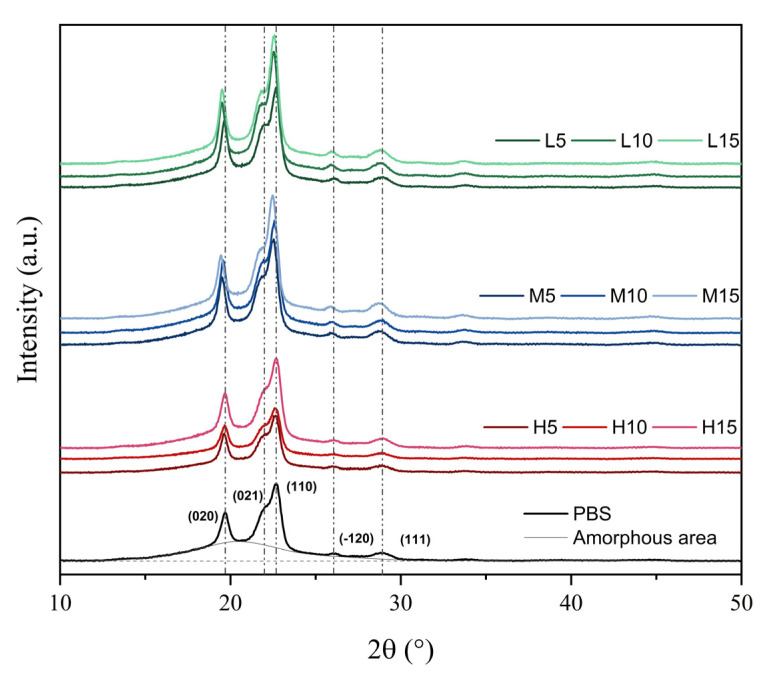
X-ray diffractograms with peak assignments of poly(butylene succinate) (PBS) with the different oligomers of butylene succinate (H, M, L series) at 5, 10, and 15 wt%.

WAXD analysis was conducted on the film samples to better elucidate the effect of the oligomers on the crystalline structure of PBS. Figure 4 shows the diffractograms, whereas Table 5 includes the percentage of crystallinity (*χ*_c_) and the unit cell parameters of PBS and PBS/OBS films.

In all the diffractograms, the five main characteristic diffraction peaks for PBS were seen, located at 19.5°, 22.0°, 22.5°, 25.9°, and 28.8°, corresponding to the (020), (021), (110), (−121), and (111) crystal planes, respectively [45,46,47]. The peak position was maintained in the oligomer-containing samples, confirming that the monoclinic α-crystal structure typical of PBS was preserved across all samples [46]. However, a small shift in the peaks was observed mainly in samples containing L-OBS and the highest ratio of OBS-M. Likewise, as also inferred from the ΔH_m_ values attained during the DSC analysis, OBS incorporation induced a significant (*p* < 0.05) increase in the degree of crystallinity of the films, which was determined through the ratio of the crystalline and amorphous areas of the diffraction spectra. Thus, crystallinity progressively increased as the OBS molecular weight decreased, but showed that the oligomer content had no significant effect (*p* > 0.05). In particular, the crystallinity increased from 41%, for the neat PBS film, up to values of 45–46% for the L-OBS-containing PBS films. As reported above, the higher crystallinity attained in the L series (M_n_ = 1150 g·mol^−1^) can be attributed to the greater promotion of molecular mobility in the PBS matrix. Similarly, the H series (M_n_ = 18,650 g·mol^−1^) and M series (Mn = 8700 g·mol^−1^) also promoted crystallinity but to a lower extent (42–43%). This further confirms the role of the molecules with very low molecular weight in the segmental mobility promotion and hence in the chain arrangement forming the crystalline domains.

As regards the cell unit parameters, a progressive decrease in the *c* parameter was observed with both the OBS molecular weight decrease and content increase. Differences among the samples were small but still significant (*p* < 0.05) for the PBS films with L-OBS and the highest content of M-OBS. This further supports the above-described phenomenon based on a subtle reorganization of the crystalline network when the end groups of the oligomers interfere with the arrangement of the PBS chains, which becomes more noticeable as the proportion of these terminal groups in the composition increases.

### 3.5. Thermomechanical Properties of PBS/OBS Films

**Figure 5:**
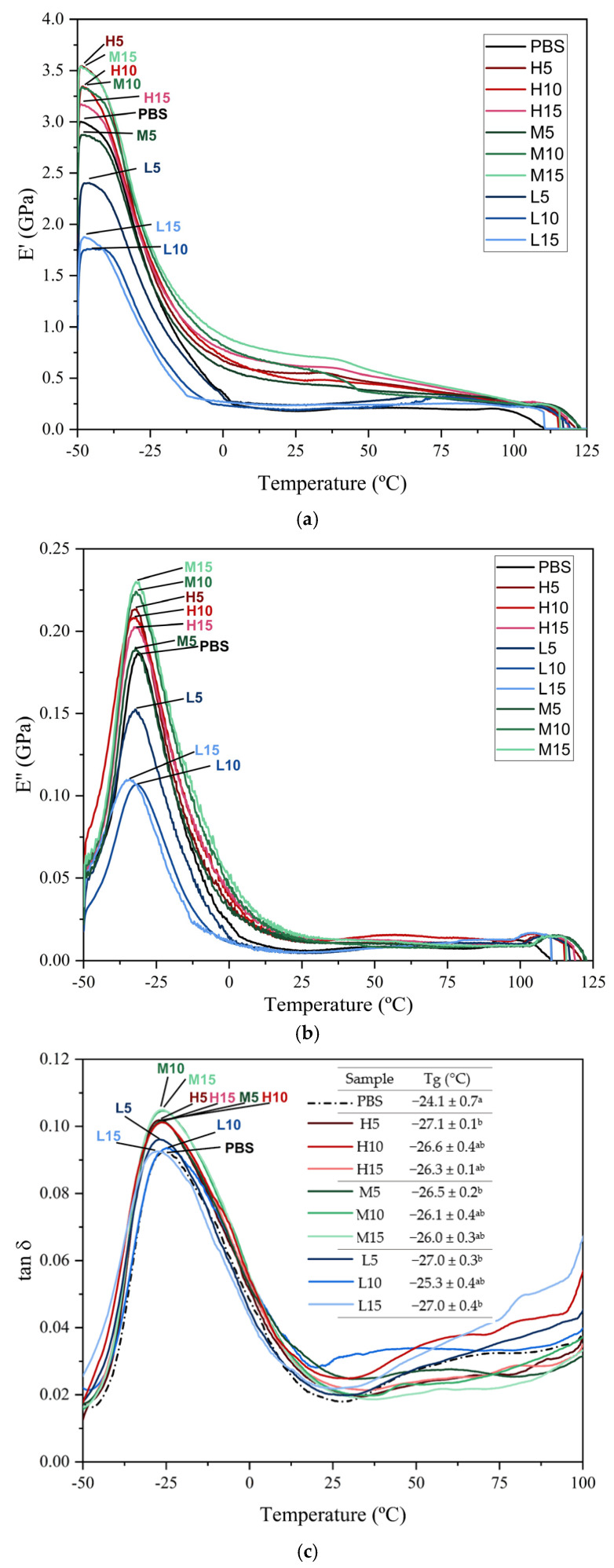
(**a**) Storage modulus (*E*′), (**b**) loss modulus (*E*″), and (**c**) damping factor (*tan δ* peak) and glass transition temperature (T_g_) of poly(butylene succinate) (PBS) films with the different oligomers of butylene succinate (H, M, L series) at 5, 10, and 15 wt%. ^a,b^ Different superscripts in the same column indicate significant differences among samples (*p* < 0.05).

The samples were further submitted to dynamo-mechanical analysis as a function of temperature to better analyze the effect on the phase transitions of PBS by the OBS incorporation. Figure 5 shows the evolution as a function of temperature of the film samples of the three main parameters: the storage modulus (E’) (Figure 5a), loss modulus (E″) (Figure 5b), and loss tangent (*tan δ*) (Figure 5c).

Neat PBS exhibited a high storage modulus (E′) at low temperatures (−50 °C), with values around 3.0 GPa, consistent with a rigid semicrystalline matrix in the glassy state. The sharp decrease in *E*’ values with the temperature increase reflects the decrease in the elastic response of the films during the glass transition of the amorphous phase. A second small step in the E’ decrease was observed above 30 °C that can be related to the non-reversible glass-to-rubbery relaxation previously described during the DSC analysis. Relative to neat PBS, the incorporation of high- and medium-molecular-weight oligomers resulted in higher values of E’ below the glass transition region, while OBS-L markedly reduced the E’ values. These differences in the E’ values can be related to the changes in the film crystallinity as well as in the different cohesion forces in the amorphous phase promoted by OBS, depending on both their molecular weight and content. Thus, for the samples prepared with the H- and M-type OBS, the crystallinity increase effect seems to overcome the contribution of the weakening of the amorphous phase, as deduced from the highest E’ values attained in the PBS films. In contrast, this weakening effect of the amorphous phase was the predominant effect for OBS-L-containing films, mainly at the highest contents (10 and 15 wt%). As commented on above, this behavior can be attributed to the short chain length of OBS-L (M_n_ = 1100 g·mol^−1^), which reduces molecular interactions and promotes free volume within the PBS matrix to a greater extent.

The evolution of the loss modulus (*E*″) as a function of temperature (Figure 5b) reflected that all the film samples exhibited a sharp peak during the *α*-transition related to the biopolymer’s *T*_g_. This thermomechanical change is proportional to the energy dissipated in the films during the loading cycle and confirms that the main glass-to-rubber transition of PBS takes place at approximately −25 °C. In terms of the oligomer effect, only the L-series exhibited reduced E″ peaks with respect to PBS, indicative of diminished segmental friction due to plasticization and reduced cohesiveness. Analysis of the loss tangent (*tan δ* peak), shown in Figure 5c, further confirmed this behavior. The evolution of the *tan δ* peak with temperature provided key insights into the viscoelastic behavior of the PBS-based systems and their α-transition dynamics. Neat PBS showed a moderate *tan δ* peak around −24 °C, consistent with the T_g_ values reported for semicrystalline PBS [48,49]. The incorporation of oligomers caused a slight but statistically significant (*p* < 0.05) shift in the *tan δ* peaks toward lower temperatures compared to neat PBS, indicating enhanced segmental mobility in the amorphous regions and less restricted molecular dynamics, which in turn explains the promotion of crystallization within the matrix. Nevertheless, the effect of the oligomer content on the T_g_ reduction was notably lower than that previously attained during the melt-mixing process. This relatively low effect can be due to the fact that a fraction of oligomers are crystallizing with the PBS molecules, which is supported by the crystallinity degree increase described above during DSC and WAXD analyses, as well as in the cell unit changes induced by the oligomer presence. The thermomechanical values described herein are similar to the DMTA patterns reported previously for PBS [50]; however, this behavior differs from other previous oligomer-containing compositions. For instance, Avolio et al. [26] observed a larger reduction in the *tan δ* peak for PLA when OLA (bearing either terminal hydroxyl or carboxylic acid groups) was incorporated at different concentrations. In this regard, one should consider that PLA is mainly an amorphous polymer; therefore, the plasticizing effect of the oligomer is more intense.

### 3.6. Mechanical Properties of PBS/OBS Films

**Figure 6:**
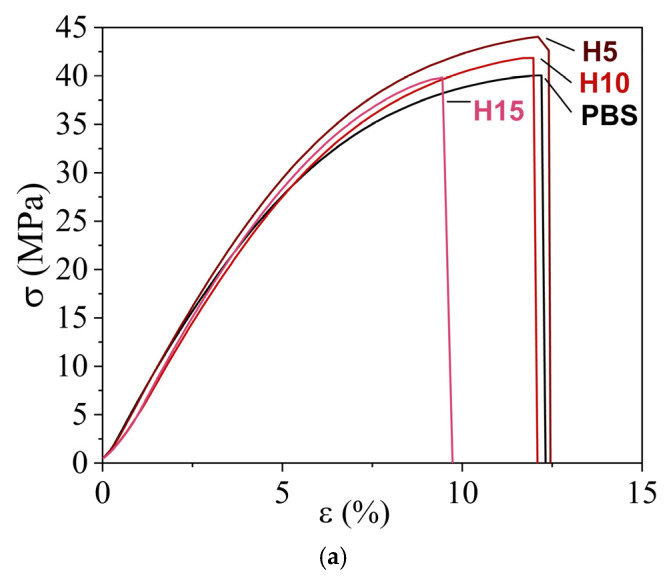

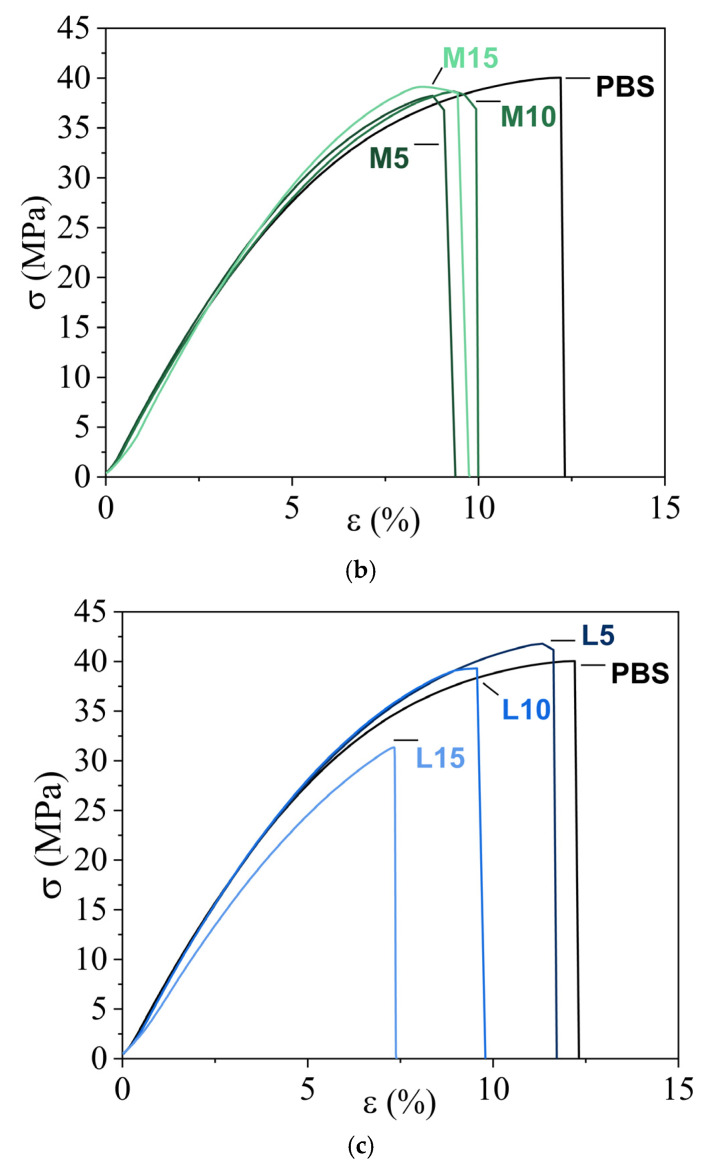
Tensile strength (*σ*) versus strain (ɛ) curves of poly(butylene succinate) (PBS) films with the different oligomers of butylene succinate: (**a**) high molecular weight (OBS-H); (**b**) medium molecular weight (OBS-M); and (**c**) low molecular weight (OBS-L) at 5, 10, and 15 wt%.

The tensile performance of the PBS and PBS/OBS films was also analyzed to ascertain the effect of the oligomers on PBS. The stress vs. strain curves of the films are shown in Figure 6, whereas the tensile parameters are summarized in Table 6.

The PBS films showed a mechanical behavior typical of rigid material with low ductility, breaking with a relatively low plastic deformation. This mechanical performance corresponds to a polymer with a high degree of crystallinity, despite the rubbery state of the amorphous phase at room temperature. For neat PBS films, the elastic modulus (*E*: 572 MPa), tensile strength at break (*σ*_max_: 39.7 MPa), and elongation at break (ɛ_b_: 12.1%) were in the range of those reported previously [26]. The PBS films containing oligomers exhibited an increase in stiffness and toughness (except the L series), with lower ductility, depending on the OBS content and its molecular weight. Thus, the ɛ_b_ values presented a progressive decrease with increasing oligomer content and decreasing molecular weight. In particular, the lowest ɛ_b_ value (7.3%) was attained for the L15 film. These results indicate that the presence of the oligomers, most notably for the L series, induces a weakening of the cohesive forces of the PBS matrix. Interestingly, the incorporation of high-molecular-weight oligomers (H series) contributed to significantly (*p* < 0.05) increase in stiffness and toughness, with the E and *σ*_max_ values increasing up to 646 and 42.4 MPa (H10 film), due to enhanced chain entanglement in crystalline domains with low plasticization of the amorphous phase. The increase in mechanical strength in the films with OBS-H, without losses of film ductility, resulted in a significant (*p* < 0.05) enhancement in toughness, which increased from 308 J/m^3^, for the neat PBS film, to 423 J/m^3^, for the H5 film.

Therefore, the tensile tests further confirmed the above-reported effect of the oligomers on the PBS structure. On the one hand, the partial inclusion of the oligomers in the crystal structure of the biopolymer and subsequent increase in crystallinity promotes stiffness and mechanical strength. On the other hand, however, their presence in the amorphous region disrupts the matrix continuity. The latter phenomenon becomes more noticeable for the higher oligomer contents. Other studies reporting the use of oligomers have also demonstrated their role as disruptors of the polymer matrix continuity. However, these studies have been mainly for PLA, in which improvements achieved in material elongation were due to its very low crystallinity and hence higher plasticizing effect of the oligomers. For example, the incorporation of low-molecular-weight PLA induced a decrease in the tensile strength and extensibility of PLA [24]. Similarly, Burgos et al. [22] described a decrease in tensile strength and elastic modulus when 15 wt% of OLAs were incorporated into PLA films. The elastic modulus decreased by up to 48% for low-molecular-weight OLAs, indicating a substantial reduction in stiffness, consistent with the matrix cohesion impairment. Ambrosio et al. [23] also reported a significant reduction in both elastic modulus and tensile strength when OLAs were incorporated into PLA films.

### 3.7. Barrier Properties of PBS/OBS

**Figure 7:**
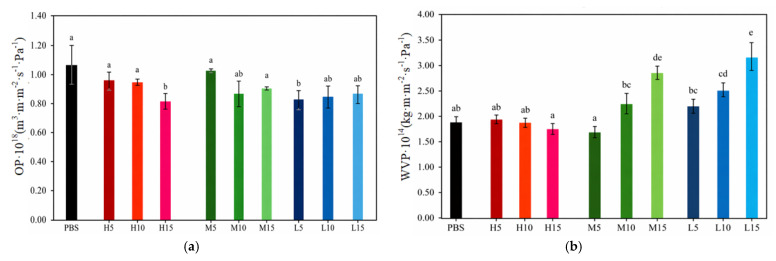
(**a**) Oxygen permeability (OP) and (**b**) permeability to water vapor permeability (WVP) of poly(butylene succinate) (PBS) films with the different oligomers of butylene succinate (H, M, L series) at 5, 10, and 15 wt%. Different letters indicate significant differences among films (*p* < 0.05).

The water vapor permeability (WVP) and oxygen permeability (OP) values of the PBS and PBS/OBS films are presented in Figure 7. Barrier performance against water vapor and oxygen is of primary relevance in food packaging applications. While water vapor resistance is crucial for preventing physical and chemical degradation due to moisture variations, oxygen barrier properties are especially important for oxidation-sensitive products such as meat, fish, or fatty foods. In this context, biopolyesters derived from succinic acid are typically characterized by moderate water vapor barrier properties and moderate-to-low resistance to oxygen transmission [34].

The neat PBS film exhibited an OP value of 1.04 × 10^−18^ m^3^·m·m^−2^·s^−1^·Pa^−1^ and a WVP value of 1.90 × 10^−14^ kg·m·m^−2^·s^−1^·Pa^−1^, which are consistent with those reported for semicrystalline PBS films [3,50]. Upon incorporation of oligomers, significant variations in the barrier performance of the PBS films were observed depending on both oligomer content and molecular weight. PBS films with high-molecular-weight oligomers (OBS-H) showed the most favorable barrier profile, particularly at the highest content. Thus, the H15 film presented the lowest OP value (8.3 × 10^−19^ m^3^·m·m^−2^·s^−1^·Pa^−1^) and one of the lowest WVP values (1.80 × 10^−14^ kg·m·m^−2^·s^−1^·Pa^−1^). In contrast, the PBS films containing low-molecular-weight oligomers (OBS-L) displayed a reverse trend. Although OP values were slightly but still significantly (*p* < 0.05) reduced with respect to neat PBS (e.g., L15 = 8.80 × 10^−19^ m^3^·m·m^−2^·s^−1^·Pa^−1^), WVP remarkably increased, with L15 exhibiting the highest value across all formulations (3.19 × 10^−14^ kg·m·m^−2^·s^−1^·Pa^−1^).

These results highlight that the oligomer incorporation modifies the PBS structure in a way that the two distinct parameters influencing the permeability, that is, solubility, a thermodynamic parameter, and diffusivity, a kinetic one, are altered. In one respect, oligomers induced an increase in the PBS crystallinity that, in turn, reduces vapor and gas molecules’ diffusivity. In particular, crystalline domains can form impermeable barriers for the diffusion process, increasing the average path length of these penetrant molecules. In the other, the presence of oligomers in the amorphous phase of PBS exerts a plasticizing effect and also disrupts the matrix continuity, which favors the transport of penetrant molecules. Furthermore, the increase in the end-chain group ratio in the macromolecule can also induce polar changes in the macromolecular composition that affect the solubility of water (increases) and oxygen (decreases). This dual effect was particularly positive in the case of the OBS-H-containing PBS films, which benefit from the crystallinity increase and are less plasticized by the oligomers. As opposite, the PBS films containing the OBS-L and OBS-M were more permeable, more notably for water vapor at the higher oligomer contents. In the latter case, the plasticization effect dominates over the crystallinity contribution, as the increased free volume and segmental mobility in the amorphous phase enhance diffusivity, outweighing the barrier effect introduced by crystalline domains. These findings are consistent with previous studies on oligomer-containing biopolymer systems [23,51], where the addition of short-chain oligomers decreased oxygen barrier modestly while markedly increasing water vapor permeability. Schliecker et al. [25] also reported that the incorporation of oligomers can enhance biopolyester film’s hydrophilicity due to the increase in the hydroxyl and carboxyl end groups.

### 3.8. Migration of PBS/OBS

**Figure 8:**
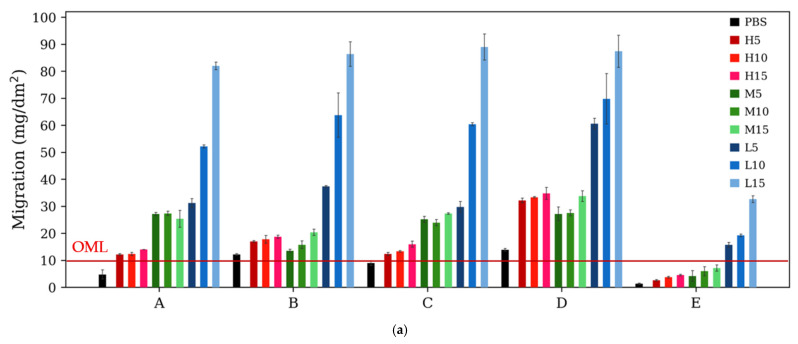

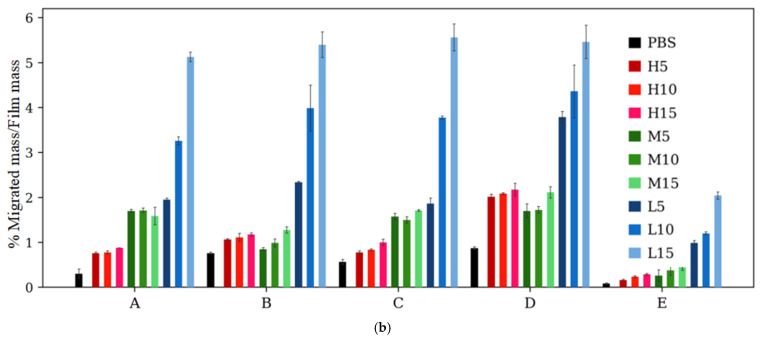
(**a**) Overall migration of poly(butylene succinate) (PBS) films with the different oligomers of butylene succinate (H, M, L series) at 5, 10, and 15 wt% tested for 10 days at 40 °C in the food simulants: (A) water; (B) 10 vol% ethanol in water; (C) 3 (wt/vol)% acetic acid in water; (D) 95 vol% ethanol in water; and (E) isooctane. (**b**) Percentage of migration expressed as mass of migrated substance (mg)/total mass of film (mg) for the different food simulants (A–E). Data are presented as mean ± standard deviation.

Finally, migration was studied due to the potential release of low-molecular-weight substances from bioplastic films and the results are gathered in Figure 8. The OM values obtained for each simulant are presented in Figure 8a. All formulations were tested for 10 days at 40 °C, conditions corresponding to the standardized test conditions OM2.

Neat PBS exhibited the lowest migration values across all simulants, ranging from 1.3 mg/dm^2^ (isooctane) to 13 mg/dm^2^ (95 vol% ethanol). Compliance with the European regulatory limit of 10 mg/dm [52] was achieved only for water, 10 vol% ethanol, and isooctane simulants. In contrast, migration values exceeded this limit in 3 (wt/vol)% acetic acid and 95 vol% ethanol, indicating that PBS may not be suitable for packaging applications involving acidic (e.g., vinegar-based) or high-fat food systems. Contradictory results have been reported in the literature regarding the migration behavior of PBS-based materials. For instance, Jariyasakoolroj et al. [53] reported migration values slightly affected by the polarity of the simulant, obtaining similar values at 40 °C in 10 vol% ethanol (2.35 mg/dm^2^) and 95 vol% ethanol (2.40 mg/dm^2^). In contrast, Ashraf et al. [54] observed a stronger dependence on simulant nature, reporting migration values of approximately 3 mg/dm^2^ and 8 mg/dm^2^ in 10 and 50 vol% ethanol, respectively, at 70 °C. These differences were attributed to the chemical structure of PBS, which contains relatively non-polar aliphatic segments that may enhance the affinity of migrating species towards lipophilic environments.

The migration behavior observed in this study was strongly dependent on the oligomer content, molecular weight, and the nature of the simulant. The incorporation of OBS oligomers significantly (*p* < 0.05) increased global migration in all film samples, particularly in the L-series, where migration reached up to 85 mg/dm^2^ in 10 vol% ethanol for the L15 sample. These trends are consistent with the higher molecular mobility of OBS-L (M_n_ = 1100 g·mol^−1^), whose migration increases with its concentration. A similar behavior was reported for PLA/OLA formulation by Avolio et al. [26]. Authors found a negligible migration for neat PLA under the same test conditions (10 days at 40 °C), which increased proportionally to the oligomer content in films. However, it is worth noting that the unmodified PLA amorphous phase remains glassy at these conditions, as opposed to PBS.

In terms of food simulant, the highest migration values were observed in the less polar simulant (95 vol% ethanol). This can be explained by the enhanced solvation capacity of ethanol and the swelling of the biopolymer matrix in contact with the simulant. The latter promotes molecular diffusion and migration of the oligomers, this being favored when their molecular weight decreases. In acidic media, diffusion of acetic acid within the matrix could enhance both the PBS swelling and the partial hydrolysis of the chains, thus increasing the overall migration values compared to pure water. In contrast, isooctane, used as a fatty food simulant, resulted in the lowest migration values across all samples. However, the formulation L15 reached the highest value of 32 mg/dm^2^, suggesting that in the L-series, oligomers can still migrate significantly under non-polar conditions, particularly when these are present at high contents.

From a structure–property perspective, the migration results correlate inversely with the molecular weight of the oligomers and directly with the polymer free volume in the amorphous phase fraction. The XRD data showed that increasing oligomer content, especially in the L-series, increased the film crystallinity, while DMA reflects the plasticization effect in the amorphous phase. The increased chain mobility in the latter phase facilitates diffusion, while the presence of low-molecular-weight molecules enhances their transport through the polymer matrix. These observations are consistent with previous studies on formulated PBS systems. For instance, Moliner et al. [55] reported significantly higher migration values in PBS-based materials containing mineral fillers (<15 wt% talc), reaching up to ~28 mg/dm^2^ in 50 vol% ethanol under similar conditions. This behavior was attributed to ethanol-induced plasticization, swelling, and hydrolytic degradation, as well as to the presence of additives of low molecular weight.

The percentage of migrated mass over film mass, represented in Figure 8b, shows a clear dependence on oligomer type and, only for the OBS-L-containing films, a strong dependence on concentration. These values, which are obviously in agreement with the trends observed in global migration, facilitate relating the amount of migrated mass to the oligomer content. In all food simulants (A–E), neat PBS exhibits the lowest migration percentages, with values always below 1%. Similarly, Poggetto et al. [56] evaluated the amount of the migrated mass with respect to PBS film mass in 10 vol% ethanol, achieving percentages of less than 0.5%. For the PBS films containing H- and M-type oligomers, this percentage was kept low, with values increasing up to approximately 2% and showing no significant differences (*p* > 0.05) as a function of oligomer concentration. In contrast, OBS-L-containing films exhibited a markedly higher migration percentage, ranging from 1 to 5%, with a clear increase as oligomer content rises. This behavior indicates that low-molecular-weight oligomers significantly enhance the release of material from the film and, from a mechanistic perspective, most of this migrated mass can be associated with oligomers present in the amorphous phase, where molecular mobility is higher. Therefore, the fact that migration remains limited to approximately 5% of the total film suggests that the majority of the material—particularly the crystalline domains—remains structurally stable and does not participate in the mass transfer phenomenon.

## 4. Conclusions

Fully bio-based OBS with varying molecular weights were homogeneously incorporated at different contents into PBS via melt mixing, and films from the resultant formulations were obtained by thermo-compression. Oligomers were able to modulate the properties of the films depending on their molecular weight and content. During melt mixing, oligomers effectively reduced viscosity and highly plasticized the PBS melt, making them promising additives as processing aids. After cooling, miscibility was maintained, resulting in a homogeneous microstructure, whereas the increased fluidity of the mixture promoted crystallization. A model was proposed based on OBS molecules distributed partially between the amorphous and crystalline phases of the PBS matrix, altering the crystal cell unit and weakening the molecular cohesion of the amorphous phase. This was further supported by the moderate decrease observed in the biopolymer’s T_g_, where only a fraction of oligomers remains in the PBS amorphous phase, and is consistent with the modification of its crystalline lattice through the slight shift in the WAXD peaks. These structural changes became more evident in the case of OBS-L due to its much smaller molecular size and the higher proportion of end-chain groups in the resultant material. Oligomers also affected the mechanical behavior. In particular, crystallinity increase promoted the stiffness and strength of the matrix, but as the content increased, the weakening effects in the amorphous phase were more noticeable, resulting in very evident effects for OBS-L with its much lower molecular weight. Oligomer incorporation also exerted a noticeable change in the gas barrier properties. On the one hand, the increased crystallinity reduced permeability by reducing diffusivity, whereas the different mobilizing effect on the amorphous phase and the polarity changes associated with the different proportion of terminal groups increased water vapor solubility and reduced oxygen solubility. Regarding migration, oligomers induced a notable increase, resulting in OM values exceeding the legal limit for all food simulants, except isooctane, then posing safety concerns. Future studies will be focused on analyzing the effect of the oligomers on the biodegradability of PBS and the use of the resulting PBS/OBS materials in the form of interlayers to be safely applied in the food packaging area.

## Figures and Tables

**Table 1:** Number- and weight-average molecular weights (M_n_ and M_w_) and dispersity index (Ð) of commercial poly(butylene succinate) (PBS) and butylene succinate oligomers with high (OBS-H), medium (OBS-M), and low molecular weights (OBS-L).

Sample	M_n_ (g·mol^−1^)	M_w_ (g·mol^−1^)	Ð
PBS	50,900	140,400	2.2
OBS-H	18,650	33,147	1.7
OBS-M	8700	16,150	1.9
OBS-L	1100	2050	1.8

**Table 2:** Formulation set of commercial poly(butylene succinate) (PBS) with butylene succinate oligomers with high (OBS-H), medium (OBS-M), and low molecular weights (OBS-L).

Sample	PBS (wt%)	OBS-H (wt%)	OBS-M (wt%)	OBS-L (wt%)	Film
PBS	100	0	0	0	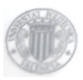
H5	95	5	0	0	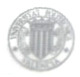
H10	90	10	0	0	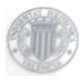
H15	85	15	0	0	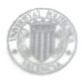
M5	95	0	5	0	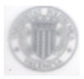
M10	90	0	10	0	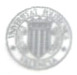
M15	85	0	15	0	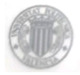
L5	95	0	0	5	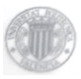
L10	90	0	0	10	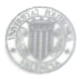
L15	85	0	0	15	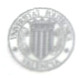

**Table 3:** Temperatures at the onset of degradation (5% weight loss: T_5%_) and maximum degradation rate (T_peak_) of poly(butylene succinate) (PBS) films with the different oligomers of butylene succinate (H, M, L series) at 5, 10, and 15 wt%.

Sample	25–450 °C	
	T_5%_ (°C)	T_peak_ (°C)
PBS	345 ± 5 ^a1^	399 ± 1 ^a1^
H5	330 ± 15 ^a12^	400 ± 1 ^a1^
H10	330 ± 8 ^a2^	399 ± 1 ^a1^
H15	326 ± 1 ^a2^	396 ± 4 ^ab1^
M5	308± 7 ^b2^	396 ± 1 ^ab2^
M10	298± 10 ^b3^	393 ± 1 ^b2^
M15	300 ± 8 ^b3^	394 ± 2 ^b1^
L5	325 ± 11 ^b2^	400 ± 1 ^a1^
L10	318± 4 ^b2^	399 ± 1 ^a1^
L15	306 ± 1 ^c3^	397 ± 1 ^ab1^

Different superscripts within the same column denote statistically significant differences (*p* < 0.05) among films containing the same oligomer at different concentrations (a–c), and among films with the same concentration and different oligomers (1–3).

**Table 4:** Melting temperature (*T*_m_), enthalpy of melting (ΔH_m_), and crystallization temperature (*T*_c_) of films of poly(butylene succinate) (PBS) with the different oligomers of butylene succinate (H, M, L series) at 5, 10, and 15 wt%.

Sample	First Heating		Cooling	Second Heating	
	T_m_ (°C)	∆H_m_ (J/g)	T_c_ (°C)	T_m2_ (°C)	∆H_m_ (J/g)
PBS	114.2 ± 1.3 ^a1^	70.2 ± 0.2 ^d3^	85.2 ± 0.2 ^a1^	114.0 ± 0.1 ^a1^	72.3 ± 4.4 ^c3^
H5	115.1 ± 1.7 ^a1^	81.8 ± 0.4 ^b1^	84.3 ± 0.1 ^b2^	113.3 ± 0.6 ^a1^	80.2 ± 0.7 ^b2^
H10	113.7 ± 0.5 ^a1^	79.3 ± 1.3 ^c2^	84.9 ± 0.4 ^a1^	113.5 ± 0.1 ^a1^	77.2 ± 2.2 ^c3^
H15	113.9 ± 0.1 ^a12^	76.2 ± 2.1 ^c2^	84.9 ± 0.2 ^a1^	113.2± 0.6 ^a2^	73.8 ± 0.9 ^c3^
M5	113.4 ± 0.8 ^a1^	78.0 ± 2.5 ^bc2^	84.4 ± 0.2 ^b2^	112.1 ± 0.2 ^a1^	79.3 ± 0.7 ^b2^
M10	113.9 ± 1.0 ^a1^	80.0 ± 0.9 ^b2^	84.8 ± 0.2 ^b1^	113.3 ± 0.6 ^a12^	80.4 ± 0.5 ^b1^
M15	113.4 ± 0.1 ^a12^	81.0 ± 2.5 ^b2^	84.5 ± 0.1 ^b2^	112.8 ± 0.1 ^a2^	81.5 ± 0.4 ^b1^
L5	113.2 ± 0.9 ^ab1^	79.6 ± 1.6 ^bc2^	83.5 ± 0.6 ^c3^	112.1± 0.2 ^b1^	79.6 ± 1.6 ^b2^
L10	112.5 ± 0.1 ^b2^	81.7 ± 1.0 ^b2^	81.8 ± 0.3 ^d2^	111.7 ± 0.1 ^b2^	83.4 ± 0.3 ^a1^
L15	111.8 ± 0.3 ^b2^	85.4 ± 1.8 ^a1^	80.9 ± 0.1 ^d3^	111.6 ± 0.4 ^b2^	83.4 ± 1.1 ^a1^

Different superscripts within the same column denote statistically significant differences (*p* < 0.05) among films containing the same oligomer at different concentrations (a–d), and among films with the same concentration and different oligomers (1–3).

**Table 5:** Percentage of crystallinity (*χ*_c_) and unit cell parameters of poly(butylene succinate) (PBS) with the different oligomers of butylene succinate (H, M, L series) at 5, 10, and 15 wt%.

Sample	Xc (%)	a (nm)	b (nm)	c (nm)	β (°)
PBS	41.1 ± 0.9 ^b2^	0.4258 ± 0.0033 ^a1^	0.902 ± 0.003 ^c3^	0.910 ± 0.002 ^a1^	119.9 ± 0.8 ^a1^
H5	42.0 ± 0.8 ^b2^	0.4236 ± 0.0001 ^a1^	0.904 ± 0.000 ^c3^	0.910 ± 0.002 ^a1^	119.1 ± 0.3 ^a12^
H10	43.0 ± 0.6 ^b2^	0.4226 ± 0.0013 ^a1^	0.903 ± 0.001 ^c3^	0.902 ± 0.009 ^a1^	118.8 ± 0.1 ^ab2^
H15	43.3 ± 0.9 ^b12^	0.4218 ± 0.0021 ^a12^	0.902 ± 0.001 ^c3^	0.901 ± 0.009 ^a12^	119.0 ± 0.3 ^a1^
M5	42.1 ± 0.2 ^ab2^	0.4236 ± 0.0033 ^a1^	0.910 ± 0.002 ^ab1^	0.903 ± 0.009 ^a1^	118.7 ± 0.9 ^ab12^
M10	43.4 ± 0.3 ^a12^	0.4223 ± 0.0032 ^a1^	0.907 ± 0.002 ^b2^	0.901 ± 0.003 ^a1^	119.0 ± 0.8 ^ab12^
M15	42.7 ± 0.3 ^a12^	0.4202 ± 0.0001 ^a2^	0.912 ± 0.001 ^a1^	0.894 ± 0.001 ^c2^	117.9 ± 0.1 ^c3^
L5	45.7 ± 0.9 ^a1^	0.4204 ± 0.0006 ^a1^	0.902 ± 0.001 ^c2^	0.897 ± 0.001 ^b2^	118.5 ± 0.2 ^b12^
L10	45.5 ± 0.9 ^a1^	0.4219 ± 0.0023 ^a1^	0.911 ± 0.001 ^a1^	0.892 ± 0.001 ^c2^	118.3 ± 0.7 ^ab2^
L15	44.8 ± 0.8 ^a1^	0.4236 ± 0.0011 ^a12^	0.910 ± 0.001 ^a1^	0.895 ± 0.009 ^b2^	118.9 ± 0.3 ^ab1^

Different superscripts within the same column denote statistically significant differences (*p* < 0.05) among films containing the same oligomer at different concentrations (a–c), and among films with the same concentration and different oligomers (1–3).

**Table 6:** Elastic modulus (E), maximum tensile strength (σ_max_), and elongation at break (ɛ_b_) of poly(butylene succinate) (PBS) films with the different oligomers of butylene succinate (H, M, L series) at 5, 10, and 15 wt%.

Sample	E (MPa)	σ_max_ (MPa)	ɛ_b_ (%)	Toughness (J/m^3^)
PBS	611 ± 9 ^a1^	39.7 ± 0.6 ^b2^	12.1 ± 0.9 ^a1^	308 ± 28 ^a1^
H5	621 ± 13 ^a1^	41.5 ± 1.2 ^a1^	12.9 ± 1.3 ^a1^	350 ± 32 ^a1^
H10	614 ± 25 ^a1^	42.4 ± 1.0 ^a1^	11.9 ± 0.7 ^a1^	327 ± 33 ^a1^
H15	606 ± 28 ^a1^	37.8 ± 1.4 ^c2^	9.4 ± 0.6 ^b2^	219 ± 19 ^d2^
M5	595 ± 20 ^a1^	39.0 ± 0.5 ^bc2^	10.1 ± 0.7 ^b2^	247 ± 22 ^c2^
M10	585 ± 27 ^a2^	38.8 ± 0.4 ^c23^	9.8 ± 0.7 ^b2^	239 ± 17 ^c2^
M15	608 ± 13 ^a1^	38.6 ± 0.7 ^c2^	9.3 ± 0.4 ^b2^	224 ± 15 ^c2^
L5	583 ± 17 ^a2^	40.9 ± 1.1 ^a1^	11.0 ± 0.4 ^a12^	266 ± 42 ^ab12^
L10	605 ± 33 ^a1^	38.2 ± 0.7 ^b3^	9.7 ± 0.3 ^b2^	220 ± 14 ^b2^
L15	538 ± 25 ^b2^	32.3 ± 0.7 ^a4^	7.3 ± 0.3 ^c3^	146 ± 14 ^d3^

Different superscripts within the same column denote statistically significant differences (*p* < 0.05) among films containing the same oligomer at different concentrations (a–d), and among films with the same concentration and different oligomers (1–4).

## Data Availability

The original contributions presented in this study are included in the article. Further inquiries can be directed to the corresponding author.

## Supplementary Materials

The following supporting information can be downloaded at: https://www.mdpi.com/article/10.3390/polym18101190/s1, Figure S1: Thermogravimetric analysis (TGA) and (b) first derivative analysis (DTGA) curves of the films of poly(butylene succinate) (PBS) with oligomers of butylene succinate (H, M, L series) with the different oligomers of butylene succinate (H, M L series) at 5, 10, and 15 wt%; Figure S2: Differential scanning calorimetry (DSC) thermograms corresponding to the (a) first heating, (b) cooling, and (c) second heating of the poly(butylene succinate) (PBS) films with the different oligomers of butylene succinate (H, M, L series) at 5, 10, and 15 wt%.
