# Supplementary material for: Incorporation of Fully Bio-Based Butylene Succinate Oligomers into Poly(butylene succinate) by Melt Mixing
Source: Polymers (Basel). 2026 May 13;18(10):1190. doi: 10.3390/polym18101190 (PMC13211161; doi:10.3390/polym18101190)
Supplement: Supplementary file 1 [file polymers-18-01190-s001.zip › polymers-4234466-supplementary.pdf]

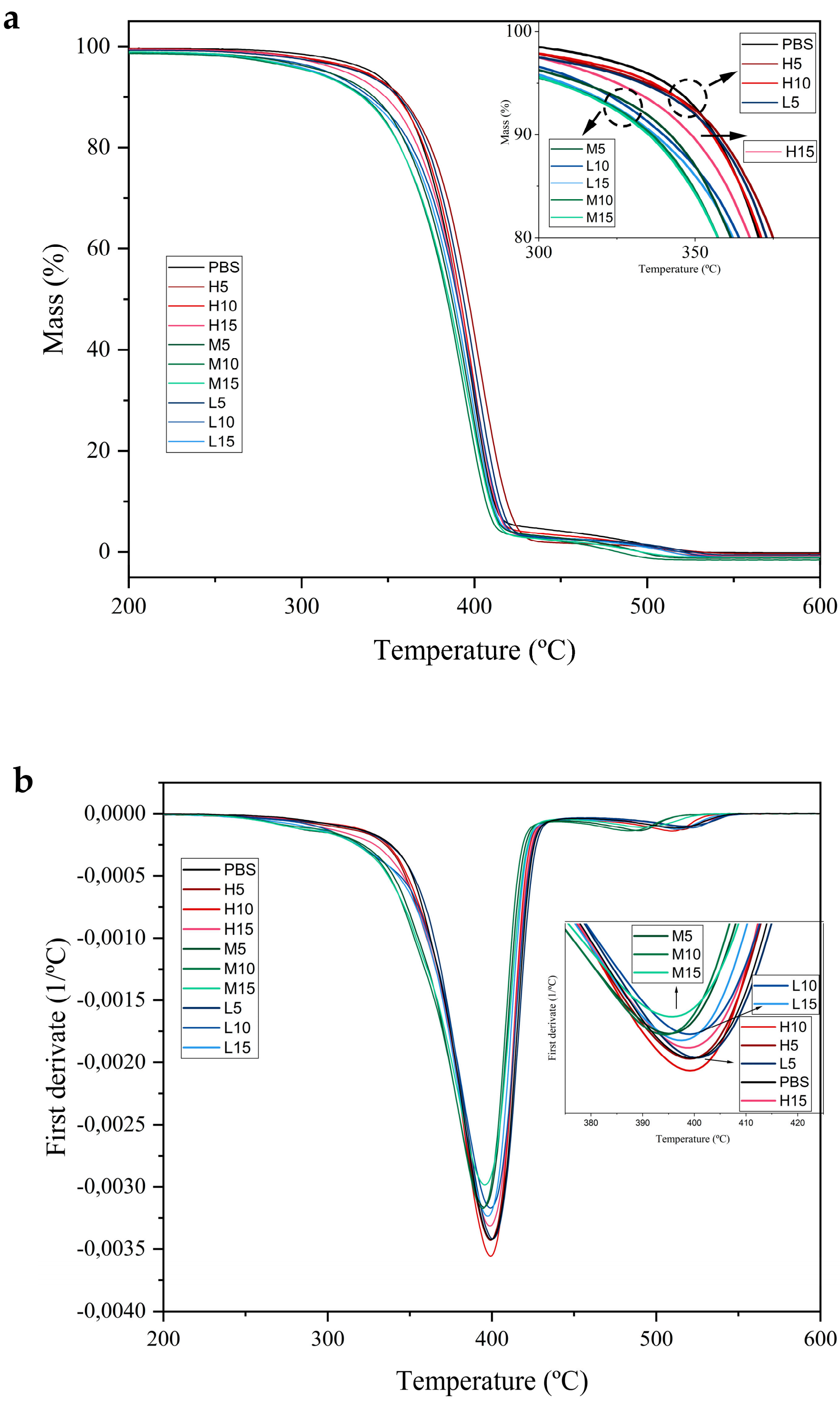

Figure S1. Thermogravimetric analysis (TGA) and (**b**) first derivative analysis (DTGA) curves of the films of poly(butylene succinate) (PBS) with the different oligomers of butylene succinate (H, M, L series) at 5, 10, and 15 wt%.

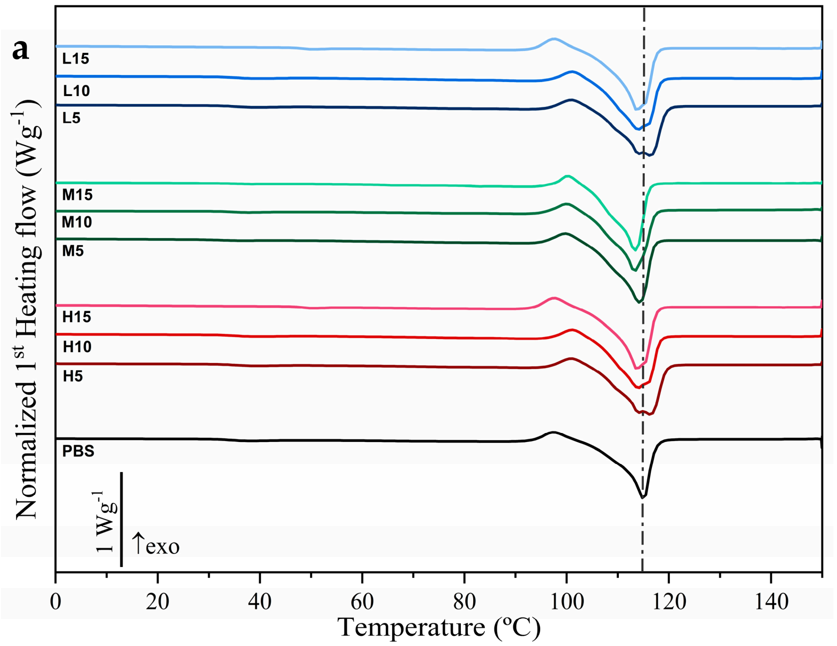
a
L15
L10
L5
M15
M10
M5
H15
H10
H5
PBS
1 Wg-1
↑exo
Normalized 1st Heating flow (Wg-1)
0
20
40
60
80
100
120
140
Temperature (ºC)

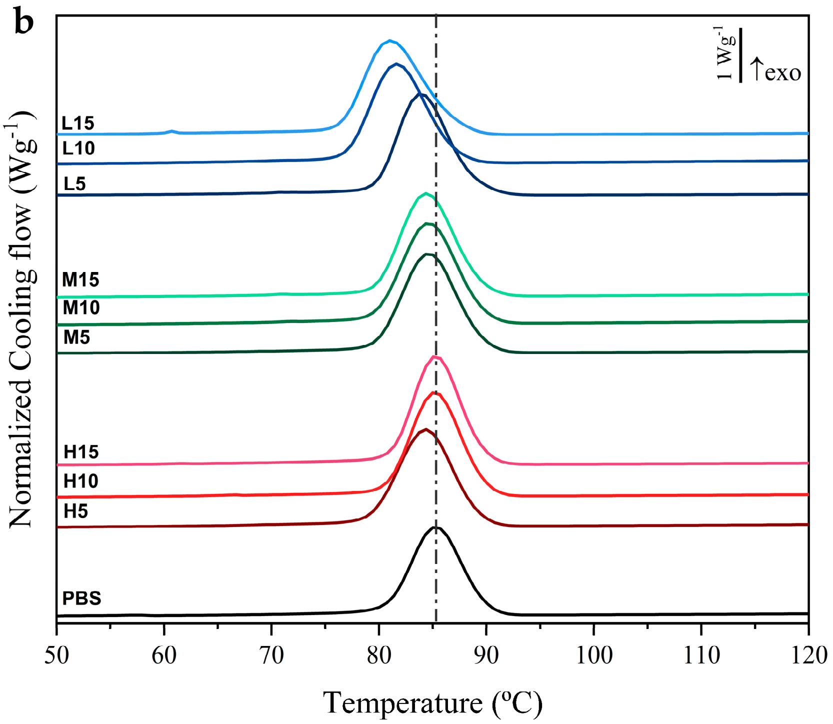
b
1 Wg-1
↑exo
L15
L10
L5
M15
M10
M5
H15
H10
H5
PBS
Normalized Cooling flow (Wg-1)
50
60
70
80
90
100
110
120
Temperature (ºC)

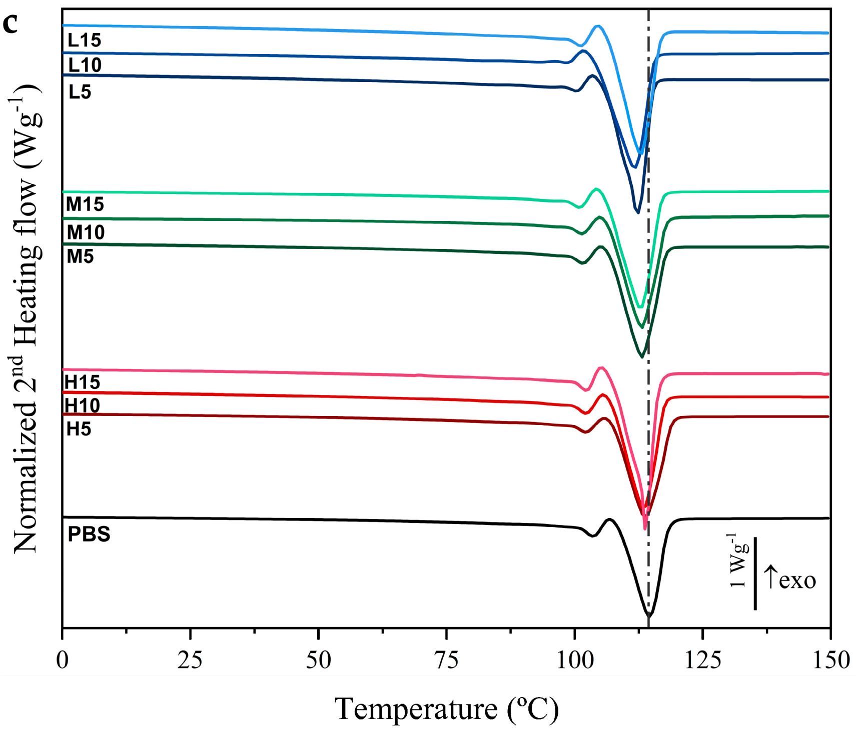

Figure S2. Differential scanning calorimetry (DSC) thermograms corresponding to the (**a**) first heating, (**b**) cooling, and (**c**) second heating of the poly(butylene succinate) (PBS) films with the different oligomers of butylene succinate (H, M, L series) at 5, 10, and 15 wt%.
